# Evaluation of Imoviral^®^ effects on early immune response in *Sparus aurata* challenged with *Vibrio anguillarum*

**DOI:** 10.3389/fimmu.2026.1681468

**Published:** 2026-04-23

**Authors:** Sabrina Natale, Serena Savoca, Fabiano Capparucci, Gioele Capillo, Monique Mancuso, Birkir Thor Bragason, Kristian Riolo, Alessia Giannetto, Fabio Marino, Carmelo Iaria

**Affiliations:** 1 Department of Chemical, Biological, Pharmaceutical and Environmental Sciences, Institute for Comparative, Experimental, Forensic and Aquatic Pathology (ICEFAP) “Slavko Bambir”, University of Messina, Messina, Italy; 2 Department of Chemical, Biological, Pharmaceutical and Environmental Sciences, University of Messina, Messina, Italy; 3 Institute for Marine Biological Resources and Biotechnology (IRBIM), National Research Council (CNR), Section of Messina, Messina, Italy; 4 National Biodiversity Future Center (NBFC), Palermo, Italy; 5 Institute for Experimental Pathology, University of Iceland, Reykjavik, Iceland

**Keywords:** acute phase response, gene expression, gilthead seabream, immunostimulant, *Vibrio anguillarum*

## Abstract

**Background:**

An acute phase response (APR) was experimentally induced in *Sparus aurata* (Linnaeus, 1758) by intraperitoneal (IP) injection of *Vibrio anguillarum* to evaluate the effects of dietary supplementation with the Imoviral^®^ complex (CRISTALFARMA) on fish growth performance and health status, through the analysis of key genes involved in the APR. Imoviral^®^ is a blend of exclusively natural extracts, i.e. uncaria *(Uncaria tomentosa)*, shiitake *(Lentinula edodes)*, beta-glucan and black-currant *(Ribes nigrum*), whose immunostimulant and analgesic properties have already been demonstrated in humans.

**Methods:**

One hundred *S. aurata* specimens 12.96 ± 0.93 grams, obtained from a fish farm, were used and divided into 5 experimental groups (in duplicate). After the feeding period, an experimental IP infection with *V. anguillarum* was performed and the APR evaluated at different time points i.e., 1, 24, 72, and 168-hours post infection. The expression of key genes involved in immune and oxidative stress responses, including *IL-1β, TNF-*α, *defensin*, *hepcidin, catalase, Copper-zinc superoxide dismutase and magnesium superoxide dismutase, Glutathione S-Transferase, TGF-β and Interleukin-10* was evaluated through RT-qPCR and compared to control groups.

**Results:**

The Imoviral^®^ diet did not affect growth performance, as all groups showed 100% survival and no significant differences in morphometric parameters. Immune-gene modulation revealed that IVS fish exhibited early and transient upregulation of TNF-α and IL-1β, while PVS fish displayed a sustained and stronger pro-inflammatory response. Antimicrobial peptides (hepcidin, defensin) were markedly overexpressed only in PVS, whereas Imoviral^®^ fed groups showed limited or temporally controlled changes. Oxidative-stress genes (CAT, CuZnSOD, MnSOD) were strongly induced in PVS, with IVS showing more moderated patterns. Cellular metabolism marker GST was significantly modulated across treatments, with IVS showing consistent differences, indicating a possibly more balanced oxidative and immune response under Imoviral^®^ supplementation.

**Conclusion:**

The present findings support the hypothesis that Imoviral^®^ may serve as a promising immunostimulant and/or antibacterial dietary supplement for farmed gilthead sea bream, providing a solid basis for future investigations in this area.

## 1 Introduction

Aquaculture is one of the fastest-growing sectors in world food production (1). Aquaculture production has seen rapid growth in terms of both production volume and economic performance over the past decades, reaching around 130.9 million tons in 2022 (2, 3). However, the rapid development of aquaculture and increased fish demand have led to the intensification of fish culture, magnifying stressors. Factors including overcrowding, handling, poor water quality, and malnutrition can lead to physiological changes in fish, such as biotic stress or immunosuppression, that increase their susceptibility to infections (4, 5). Indeed, the main factors affecting aquaculture development include infectious diseases of farmed fish, often causing high levels of mortality and partial or total loss of production. Bacterial pathogens, including *Vibrio anguillarum*, represent a major constraint for farmed teleosts, often causing significant economic losses.

Unfortunately, commonly used veterinary drugs in aquaculture, such as antibiotics and vaccines, are not a sustainable solution. In addition to high costs, antibiotics can cause several adverse effects, including the development of antibiotic resistance, immunosuppression, environmental pollution, and the accumulation of chemical residues in fish tissues which can be potentially harmful to public health (6–8). Vaccination is a valid prophylactic approach for controlling infectious diseases in fish culture; however, the development of effective formulations for several pathogens is often hampered by high production costs and the antigenic heterogeneity of microbial strains (6, 7). In recent years, due to these challenges, researchers, pharmaceutical companies, and farmers have focused on developing of alternative strategies for the managing infectious diseases in aquaculture. These strategies aim to enhance the immune responses (immunocompetence) of fish and thereby increase their resistance to pathogens. Among these strategies, the application of immunostimulants in aquaculture has emerged as one of the most promising alternatives in the prevention and control of infectious diseases (9–14). Immunostimulants are natural or synthetic compounds, with diverse origins and functions, that modulate the immune system and increase host resistance by enhancing non-specific defence mechanisms (15, 16). Several natural products, such as plant or fungi and their extracts may serve as promising immunostimulant dietary supplements that potentially aid in disease control across various organisms, including aquatic species, by upregulating host defence mechanisms against pathogens. Moreover, they can be used as a complementary approach to traditional therapies, since they provide a useful source of biologically active secondary metabolites, and are also easily available, cost- effective and environmental friendly (8, 17). Different aquaculture needs have led to the development of different methods for administering the immunostimulants, including injection, immersion, and oral administration. Among these, oral administration is the simplest and most economically feasible method for both extensive and intensive aquaculture systems. It does not induce handling stress and allows mass administration regardless of fish size; however, it can only be administered with an artificial diet. Immunostimulants that are effective in laboratory fish diets act on the non-specific immune system at different levels (18, 19). In this preliminary study, we evaluate the effects of the Imoviral^®^ complex in *Sparus aurata* fingerlings, by analyzing of the expression of selected immune-related genes involved in the acute phase response. Imoviral^®^, a commercially available for human use, is a natural formulation containing a mixture of exclusively natural extracts such as uncaria (*Uncaria tomentosa*), shiitake (*Lentinula edodes*), beta-glucan, and blackcurrant (*Ribes nigrum*), whose individual immunostimulant and immunomodulant properties have already been demonstrated in various organisms (18). The study aimed to define a starting point for assessing the potential immunomodulatory activity of Imoviral^®^ by comparing fish fed a standard commercial diet with those receiving an Imoviral^®^-supplemented diet, under both uninfected conditions and following experimental infection with *Vibrio anguillarum.* The findings from this study will provide preliminary evidence to support further investigation and potential application of the Imoviral^®^ complex in aquaculture.

## 2 Materials and methods

### 2.1 Fish maintenance, rearing conditions and experimental diet

One hundred gilthead sea bream (*Sparus aurata*) specimens (12.96 ± 0.93g) in body weight, were obtained from a local fish farm and sent to the Centre for Experimental Fish Pathology (Centro di Ittiopatologia Sperimentale della Sicilia – CISS), Department of Veterinary Sciences, University of Messina, Italy. CISS has been accredited since 2006 for use, and since 2010 for production, of aquatic organisms for experimental research (DM n°39/March/2006). In the laboratory, fish were randomly assigned to, and kept in, 10 tanks of 110 L in volume, equipped with mechanical and biological filtration, with strictly water-controlled conditions for the entire experimental trial (Temperature 20-22°C, salinity 35‰, pH 8 and dissolved oxygen (DO) 7ppm and 10L:14D photoperiod). Imoviral^®^ experimental feed was prepared by adding 0.25mg of Imoviral^®^ supplement to 10gr of commercial food (Crude Protein 46%, Crude lipids 16%, NFE% (carbohydrates) 20.6%, Ash 6.9%, Fiber 2.5%, Phosphorus (P) 1%). The dose of Imoviral^®^ (0.25 mg per 10 g of feed) was selected based on the manufacturer’s recommendations. Since no previous studies have evaluated Imoviral^®^ administration in fish, species-specific dose–response data are currently unavailable, and the manufacturer’s guidance was therefore used to define the experimental dosage. The mixture obtained was dried at room temperature for 2 hours and then ground and sieved to produce a pellet of dimensions consistent with the commercial feed of gilthead sea bream. Specimens were fed twice a day with commercial pellet (1.5% of body weight) during an acclimatization period of 45 days (14.556 ± 0.99g), prior to the experimental trial. Following the acclimatization phase, 40 specimens (4 tanks) were fed with Imoviral^®^ experimental feed (0.25 mg/10 gr of pellets) (treatment). The remaining 60 fish (6 tanks) were instead fed with commercial feed (control).

### 2.2 Growth performance

Weight and length were constantly monitored during all quarantine period and experimental trial.

Growth parameters were calculated using the following equations:

Weight gain (WG) = final fish weight (Wf) − initial fish weight (Wi).

Daily growth rate (DGR) = (Wf − Wi)/T, where T represents time of study in days.

Specific growth rate (SGR) = ((ln Wf − ln Wi/T)) × 100.

The whole feeding trial lasted 30 days.

### 2.3 Bacterial challenge

The pathogenic bacterium *Vibrio anguillarum* (serotype O1) was obtained from the Istituto Zooprofilattico Sperimentale delle Venezie (IZSVe). Prior to the experimental challenge, the virulence of the pathogenic strain was enhanced through serial *in vivo* passages in gilthead sea bream specimens. Briefly, fish were infected with a dose of 0.1 ml of bacterial suspension in saline solution (10^8^ cells/ml) and monitored for 3 days. Then, blood samples were taken from the caudal vein, spread on Marine agar (MA, Difco) and incubated at 24 °C for 24–48 hours. This procedure was repeated 3 consecutive times. The sub-lethal dose selected for bacterial infection was 10^5^ cells/ml (19–21).

At the end of the experimental feeding phase, fish were divided in 5 experimental subgroups, each consisting of 20 fish stocked in duplicate 100L tanks. The subgroups were as follow:

Group 1 (IVS): fish fed with Imoviral^®^ experimental feed and administered 0.1 ml of a suspension of virulent *V. anguillarum* by intraperitoneal injection (IP).
Group 2 (IPS): fish fed with Imoviral^®^ experimental feed and administered 0.1 ml of sterile saline solution by IP as control.
Group 3 (PVS): fish fed exclusively with commercial feed, and administered 0.1 ml of suspension of virulent *V. anguillarum* by IP.
Group 4 (PPS): fish fed with commercial feed and administered 0.1 ml of sterile saline solution by IP as control.
Group 5 (CTRL): fish fed with commercial feed that was not injected.

Experimental infection has been performed previous anesthesia with MS-222 (200mg/L, MS-222 buffered with NaHCO_3_). After the experimental infection, 5 fish from each experimental group were sacrificed at each sampling time points (1, 24, 72, and 168 hours post injection (hpi), by an overdose of anesthetic (500mg/L, MS-222 buffered with NaHCO_3_), according to (22). Organs were collected during necropsy and subsequently frozen at -80 °C. Spleen samples were used for molecular analyses.

### 2.4 RNA extraction and cDNA synthesis

Total RNA was isolated from spleen samples collected at each time point. Tissue homogenization and RNA extraction were performed using TissueLyser II (Qiagen) and the RNeasy Plus Mini Kit (Qiagen), according to manufacturer’s instructions. RNA integrity was evaluated on 1% (w/v) agarose gels and the concentration and purity verified using a Nanodrop spectophotometer (Thermo Scientific). cDNA synthesis carried out from 1 μg total RNA using the QuantiTect reverse transcription Kit (Qiagen), after gDNA wipe-out buffer treatment, as suggested in manufacturer’s instructions.

### 2.5 Real-time PCR

Analysis of the acute phase immune response in control and treated sea bream specimens was performed by qPCR to evaluate the modulation of a set of immune-related genes, including the cytokines *Interleukin 1 beta* (*IL-1β), Interleukin-10 (IL-10*), and *tumour necrosis factor alpha* (*TNF-α*), together with the antimicrobial peptides (AMPs) *β- defensin* (*def*), *hepcidin* (*hep*), *and Transforming Growth Factor-Beta (TGF-β)*. In addition, the expression of genes encoding for the antioxidant enzymes *catalase* (*CAT*), *Copper-zinc superoxide dismutase* (*CuZnSOD*), *manganese superoxide dismutase (MnSOD), and Glutathione S-Transferase (GST)* were also evaluated. qPCR reactions were performed with the Rotor-Gene Q 2plex Hrm thermocycler (Qiagen) using SYBR Green chemistry (Qiagen). For each gene, fifteen-fold diluted cDNA samples were run in duplicate together with no template and minus reverse transcriptase controls. PCR efficiency was determined as detailed by Fernandes et al. (2006) (23). Four different reference genes, elongation factor 1 alpha [EF-1α], glyceraldehyde 3-phosphate dehydrogenase [GAPDH], beta actin [β-act], and 18S ribosomal RNA [18S] were assessed and the normalization factor from the two most stable genes (calculated with the geNorm software, https://genorm.cmgg.be), β-act and 18S, was used to correct the raw target gene data as described by Giannetto et al. (2014) (24). The specificity of the reactions was confirmed from single-peak melting curves. Specific primer sets for each gene are detailed in S1, (**Supplementary Materials**).

### 2.6 Statistical analysis

Differences in morphometric data and growth parameters collected during the quarantine, pre-trial and trial between groups were analysed using one-way analysis of variance (one-way ANOVA). Gene expression data were analysed using three-way analysis of variance (two-way ANOVA), after checking the assumptions of normality and homoscedasticity, followed by a *post-hoc* Tukey’s Honestly Significant Difference (HSD) test, to determine eventual significant differences in gene expression in response to dietary treatments at different time points. The significance level was set at p< 0.05. Statistical analyses were conducted using Sigmaplot V.12. Two- way ANOVA data are included in S2.

## 3 Results

To elucidate the immunostimulant potential of Imoviral^®^ as a dietary additive, the expression of immune-related genes involved in the acute phase response was investigated in the spleen of *Sparus aurata* fingerlings.

### 3.1 Growth performance

During the experimental trial, all fish showed a survival rate of 100% and a constant growth in all groups. Morphometric data (weight and total length) of specimens collected during quarantine and throughout the experimental trial were expressed as mean and standard deviation and summarized in S3 and S4. Growth parameters analysis did not show any significant differences among groups (p>0.05).

### 3.2 Immune and inflammatory response

The pro-inflammatory cytokine *TNF-α* was significantly upregulated at 1, 24 and 72 hpi in Imoviral^®^-fed group IVS compared to the control group (CTRL), although no significant difference was detected at T168. Between the groups infected with *Vibrio anguillarum*, a gene expression trend was observed as follows: at 1 hpi, the IVS group showed the highest expression level of *TNF-α* (p<0.05), which was significantly different from the levels expressed in PVS and in all other groups (p<0.05) at that timepoint. At 24 hpi, *TNF-a* levels were not different (p>0.05) in IVS and PVS groups. A decrease in *TNF-α* gene expression was observed between T72 and T168 in IVS in correlation with increasing infection time (p<0.05).

**Figure 1:**
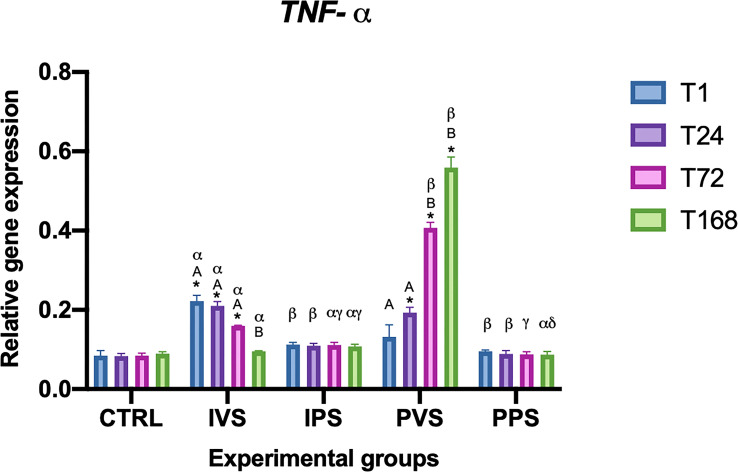
Relative expression of TNF-α mRNA in the spleen of *Sparus aurata* during experimental diet administration. Data are presented as mean ± SD (n = 5). Asterisks (*) indicate significant differences between each experimental group and the control group (CTRL) at the same time point. Letters are shown only when statistically significant differences are detected. Different capital letters indicate significant differences among time points within the same treatment group, whereas different Greek letters indicate significant differences among treatment groups at the same time point (*p* < 0.05). IVS, fish fed Imoviral^®^ and infected with *Vibrio anguillarum*; IPS, fish fed Imoviral^®^ and uninfected; PVS, fish fed commercial feed and infected with *Vibrio anguillarum*; PPS, fish fed commercial feed and uninfected; CTRL, control group.

On the contrary, *TNF-α* mRNA expression levels increased from T1-T24 to T72-T168 in the PVS group (p<0.05). When the uninfected groups were compared (IPS, fed with Imoviral^®^, and PPS, fed only with commercial feed) a significant difference was only observed at 168 hpi (p<0.05). Notably, the IPS and PPS groups did not show significant differences in *TNF-α* gene expression levels among time points within the same experimental group (**Figure 1**).

**Figure 2:**
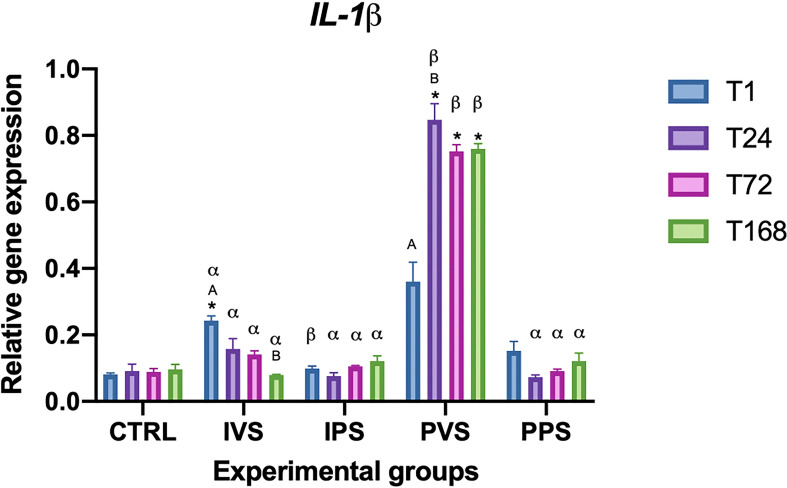
Relative expression of IL-1β mRNA in the spleen of *Sparus aurata* during experimental diet administration. Data are presented as mean ± SD (n = 5). Asterisks (*) indicate significant differences between each experimental group and the control group (CTRL) at the same time point. Letters are shown only when statistically significant differences are detected. Different capital letters indicate significant differences among time points within the same treatment group, whereas different Greek letters indicate significant differences among treatment groups at the same time point (*p* < 0.05). IVS, fish fed Imoviral^®^ and infected with *Vibrio anguillarum*; IPS, fish fed Imoviral^®^ and uninfected; PVS, fish fed commercial feed and infected with *Vibrio anguillarum*; PPS, fish fed commercial feed and uninfected; CTRL, control group.

IL-1β mRNA expression levels showed higher values in the PVS group. At T1, the only differences were observed between IVS and CTRL and between the IVS and IPS groups (p< 0.05). IL-1β gene expression was upregulated at 1 hpi in IVS, compared to CTRL, but decreased from T24 to T168, showing no significant variations with CTRL or with the other experimental groups (p>0.05), except for the PVS group. The highest level of IL-1β gene expression was observed in the PVS group in which the gene expression was higher than in all the other experimental groups (p<0.05), with maximum expression at 24 hpi from which there was a moderate decrease at 72 hpi (p<0.05). The uninfected group did not show remarkable (p>0.05) (**Figure 2**).

**Figure 3:**
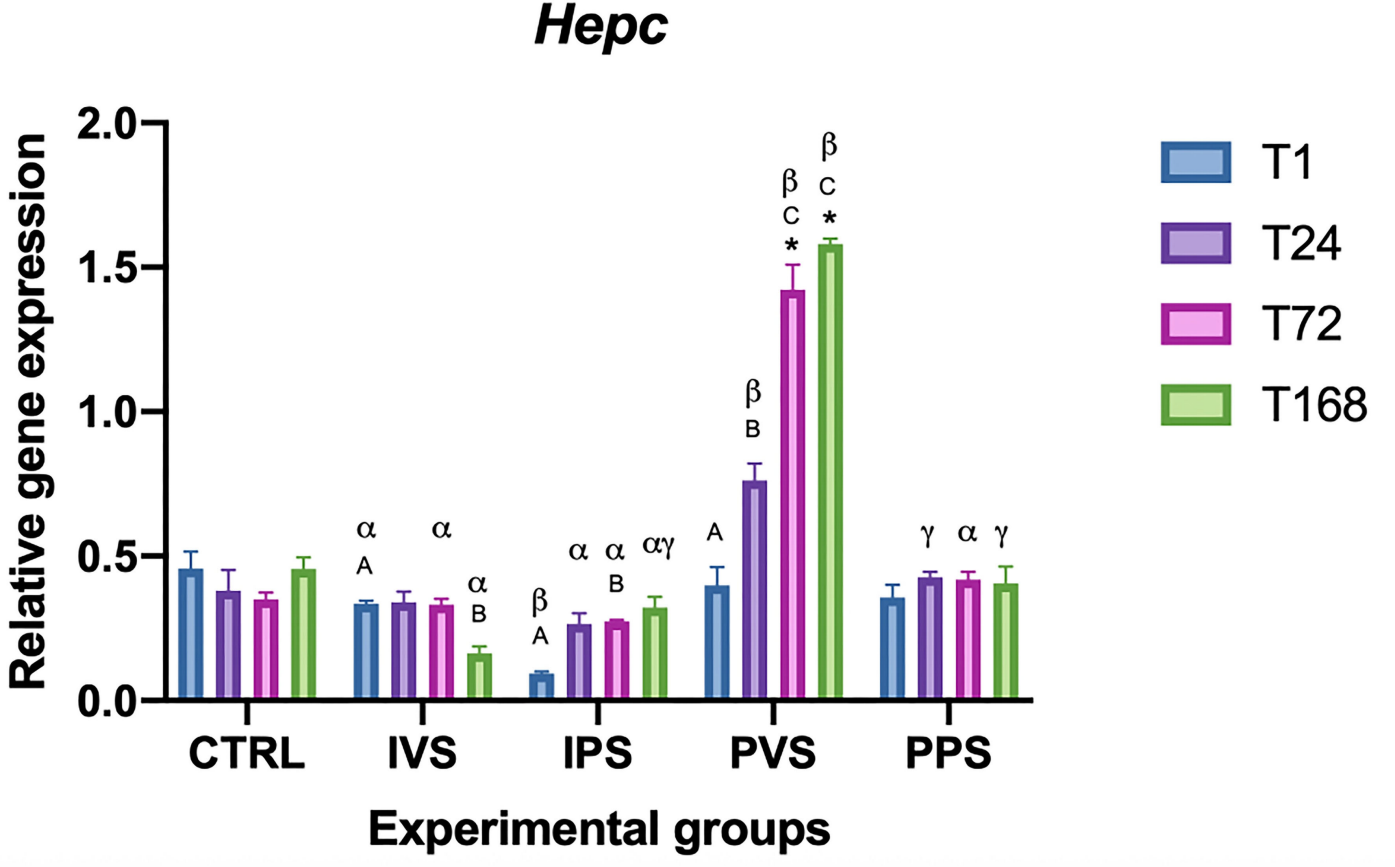
Relative expression of HEP mRNA in the spleen of *Sparus aurata* during experimental diet administration. Data are presented as mean ± SD (n = 5). Asterisks (*) indicate significant differences between each experimental group and the control group (CTRL) at the same time point. Letters are shown only when statistically significant differences are detected. Different capital letters indicate significant differences among time points within the same treatment group, whereas different Greek letters indicate significant differences among treatment groups at the same time point (*p* < 0.05). IVS, fish fed Imoviral^®^ and infected with *Vibrio anguillarum*; IPS, fish fed Imoviral^®^ and uninfected; PVS, fish fed commercial feed and infected with *Vibrio anguillarum*; PPS, fish fed commercial feed and uninfected; CTRL, control group.

Regarding the antimicrobial peptides, hepc expression was markedly upregulated exclusively in PVS (at 72 and 168 hpi) compared to CTRL (p<0.05), while none of the other groups showed significant variations compared to the control (p>0.05). At T1, only the experimental groups fed Imoviral, IVS (infected) *vs* IPS (uninfected), showed significant differences (p<0.05). At T24 IPS, PVS, and PPS varied significantly, showing the lowest values in IPS, and the highest in PVS. At T72 hepc expression follows a noteworthy trend; the two experimental groups fed Imoviral show no evident variations (IVS and IPS, p>0.05), but hepc expression shows a peak in PVS (p<0.05), the group fed commercial feed. At T168, Hepc expression levels were lower in the IVS group compared to all other groups (p<0.05), and strongly expressed in PVS (p<0.05). No significant differences were observed between the IPS and PPS groups (p> 0.05). The IVS and PVS groups were the only one to show significant changes in hepc expression over time (p < 0.05). Within the IVS group, hepc decreased significantly only at T168 compared to all other T and PVS and PPS groups (p < 0.05) (**Figure 3**).

**Figure 4:**
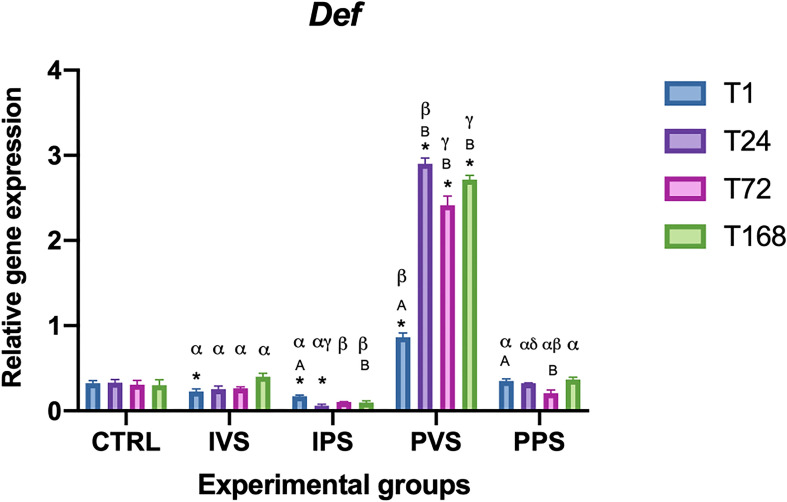
Relative expression of DEF mRNA in the spleen of *Sparus aurata* during experimental diet administration. Data are presented as mean ± SD (n = 5). Asterisks (*) indicate significant differences between each experimental group and the control group (CTRL) at the same time point. Letters are shown only when statistically significant differences are detected. Different capital letters indicate significant differences among time points within the same treatment group, whereas different Greek letters indicate significant differences among treatment groups at the same time point (*p* < 0.05). IVS, fish fed Imoviral^®^ and infected with *Vibrio anguillarum*; IPS, fish fed Imoviral^®^ and uninfected; PVS, fish fed commercial feed and infected with *Vibrio anguillarum*; PPS, fish fed commercial feed and uninfected; CTRL, control group.

Def expression varied significantly between CTRL and IVS (T1), IPS (T1, T24) and PVS (T1, 24, 72, and 168) experimental groups (p<0.05). At 1 and 24 hpi, IVS and IPS did not show significant differences in def expression levels; in both groups it was strongly decreased compared to the PVS group (p<0.05). Even PPS showed different gene expression levels compared to PVSand IPS (p<0.05). At 72 and 168 hpi, similar differences described for T24 between the experimental groups were recorded, except for IVS and IPS showing a different response at these time points. The IVS group did not show significant changes over time (p>0.05), while IPS showed significant changes only between T1 and T24. The PVS group showed significant differences between T1 and all other T (p <0.05), while PPS highlighted a significant variation between T1 and T72 (p<0.05) (**Figure 4**).

**Figure 5:**
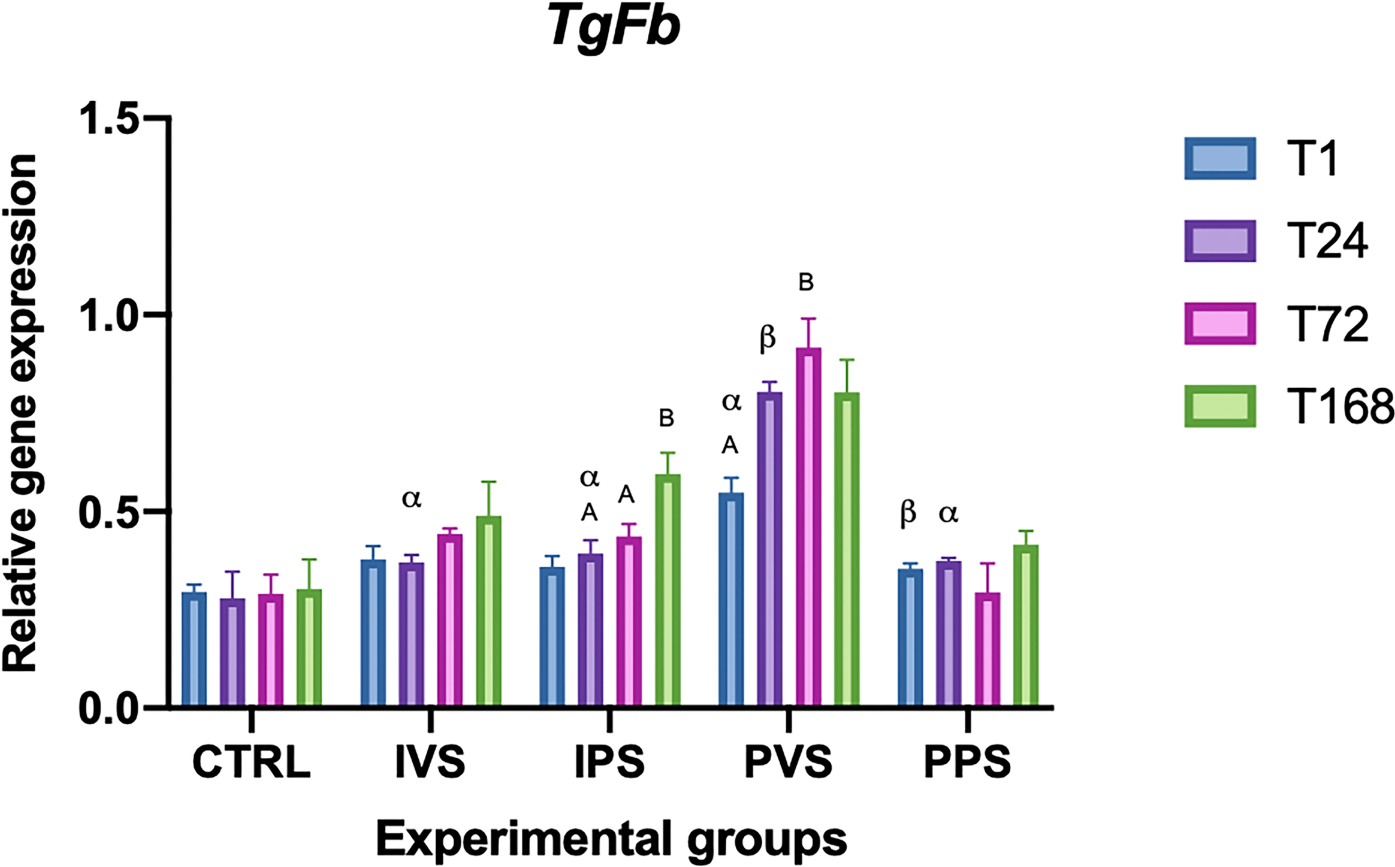
Relative expression of TGF-β mRNA in the spleen of *Sparus aurata* during experimental diet administration. Data are presented as mean ± SD (n = 5). Asterisks (*) indicate significant differences between each experimental group and the control group (CTRL) at the same time point. Letters are shown only when statistically significant differences are detected. Different capital letters indicate significant differences among time points within the same treatment group, whereas different Greek letters indicate significant differences among treatment groups at the same time point (*p* < 0.05). IVS, fish fed Imoviral^®^ and infected with *Vibrio anguillarum*; IPS, fish fed Imoviral^®^ and uninfected; PVS, fish fed commercial feed and infected with *Vibrio anguillarum*; PPS, fish fed commercial feed and uninfected; CTRL, control group.

Regarding TgFb expression, no statistically significant differences were observed comparing the treatment groups with controls (p > 0.05). However, significant changes over time were observed within some experimental groups: IPS showed differences at T24 and T168, as well as at T72 and T168, while PVS showed differences at T1 and T72 (p<0.05). Considering the same time point, significant differences were noted between PVS and PPS groups at T1. The same was true for IVS, IPS and PPS groups that showed significant differences with PVS at T24 (p<0.05) (**Figure 5**).

**Figure 6:**
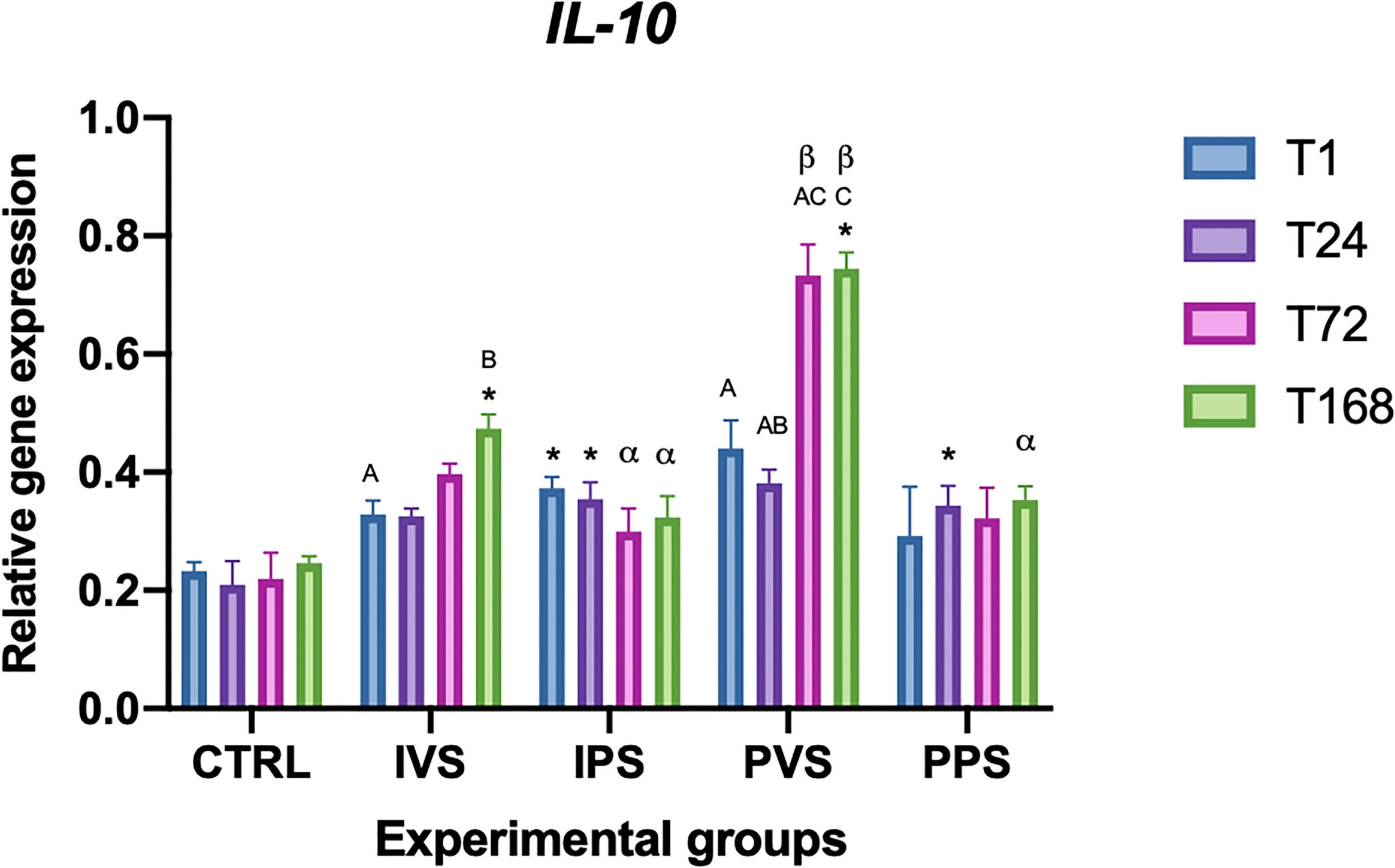
Relative expression of IL-10 mRNA in the spleen of *Sparus aurata* during experimental diet administration. Data are presented as mean ± SD (n = 5). Asterisks (*) indicate significant differences between each experimental group and the control group (CTRL) at the same time point. Letters are shown only when statistically significant differences are detected. Different capital letters indicate significant differences among time points within the same treatment group, whereas different Greek letters indicate significant differences among treatment groups at the same time point (*p* < 0.05). IVS, fish fed Imoviral^®^ and infected with *Vibrio anguillarum*; IPS, fish fed Imoviral^®^ and uninfected; PVS, fish fed commercial feed and infected with *Vibrio anguillarum*; PPS, fish fed commercial feed and uninfected; CTRL, control group.

The expression of Il-10 varied significantly between the CTRL and experimental groups (p<0.05). More specifically, both IVS and PVS group showed significant differences with controls at T168. IPS showed differences with control at T1 and T24, while PPS only at T24. The expression of Il-10 showed a significant difference over time within the same group. Differences were detected between T1 and T168 in the IVS group, and among all the time points of PVS groups, except between T1 and T24, and T1 and T72 (p>0.05). Regarding the differences between the experimental groups at the same time point, we observed a significance at T72 between the IPS and PVS groups, and IPS and PPS groups showed significant difference compared with PVS at T168 (p<0.05) (**Figure 6**).

### 3.3 Genes involved in oxidative stress response

**Figure 7:**
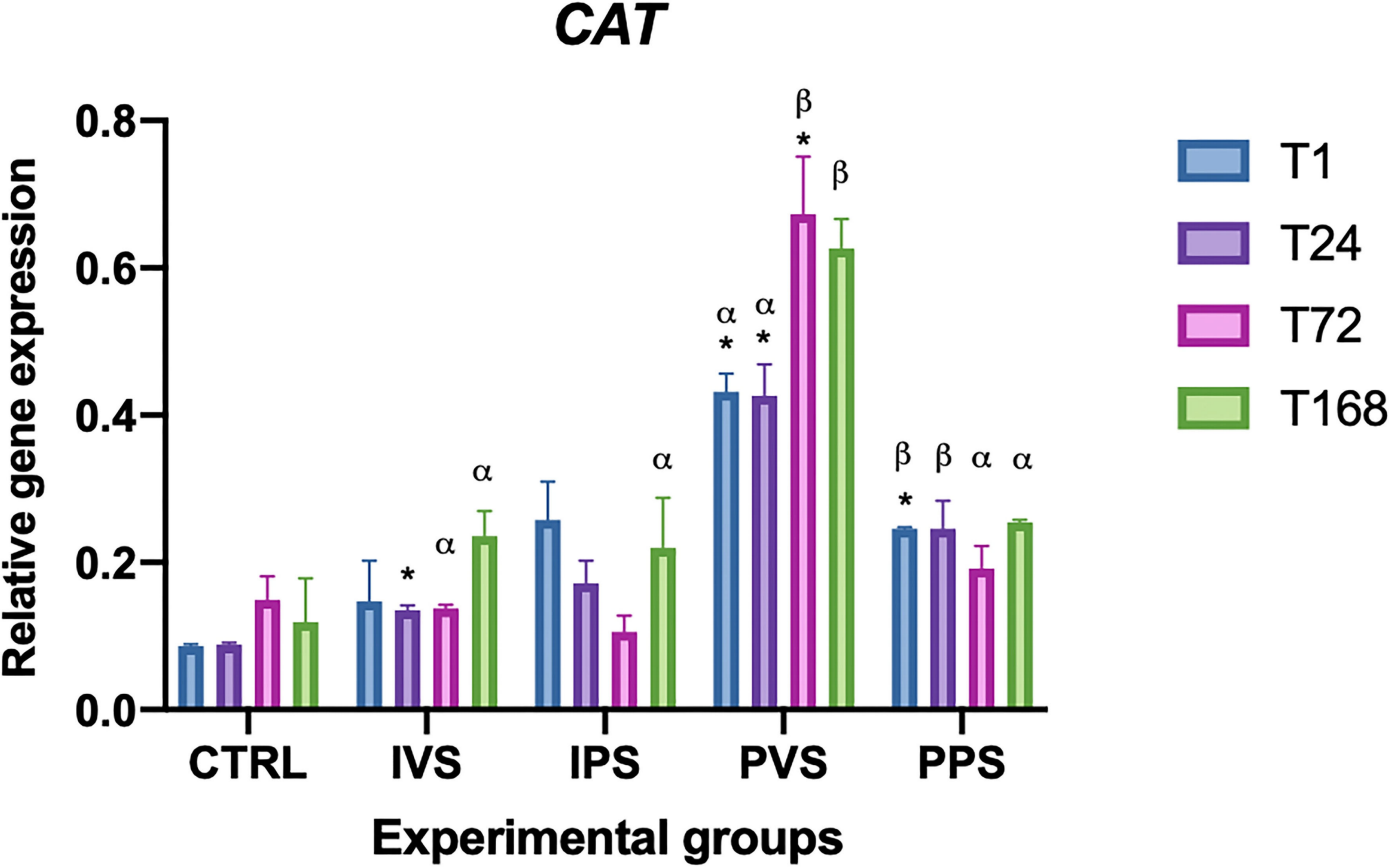
Relative expression of CAT mRNA in the spleen of *Sparus aurata* during experimental diet administration. Data are presented as mean ± SD (n = 5). Asterisks (*) indicate significant differences between each experimental group and the control group (CTRL) at the same time point. Letters are shown only when statistically significant differences are detected. Different capital letters indicate significant differences among time points within the same treatment group, whereas different Greek letters indicate significant differences among treatment groups at the same time point (*p* < 0.05). IVS, fish fed Imoviral^®^ and infected with *Vibrio anguillarum*; IPS, fish fed Imoviral^®^ and uninfected; PVS, fish fed commercial feed and infected with *Vibrio anguillarum*; PPS, fish fed commercial feed and uninfected; CTRL, control group.

*CAT* transcript levels showed a moderate increasing trend over time, with a marked peak at 72 and 168 hpi in PVS group. Significant differences between controls and experimental groups were observed at T24 for IVS, and at T1 for PPS. The same was observed for PVS at T1, T24, and T72 (p<0.05), while IPS groups showed no significant differences between controls and treatment group. Regarding the differences between experimental groups at the same time point, CAT gene expression was statistically different between PVS and PPS groups in all the time points, while the comparison between IVS and PVS showed significant difference in CAT transcript levels at T72 and T168 (**Figure 7**).

**Figure 8:**
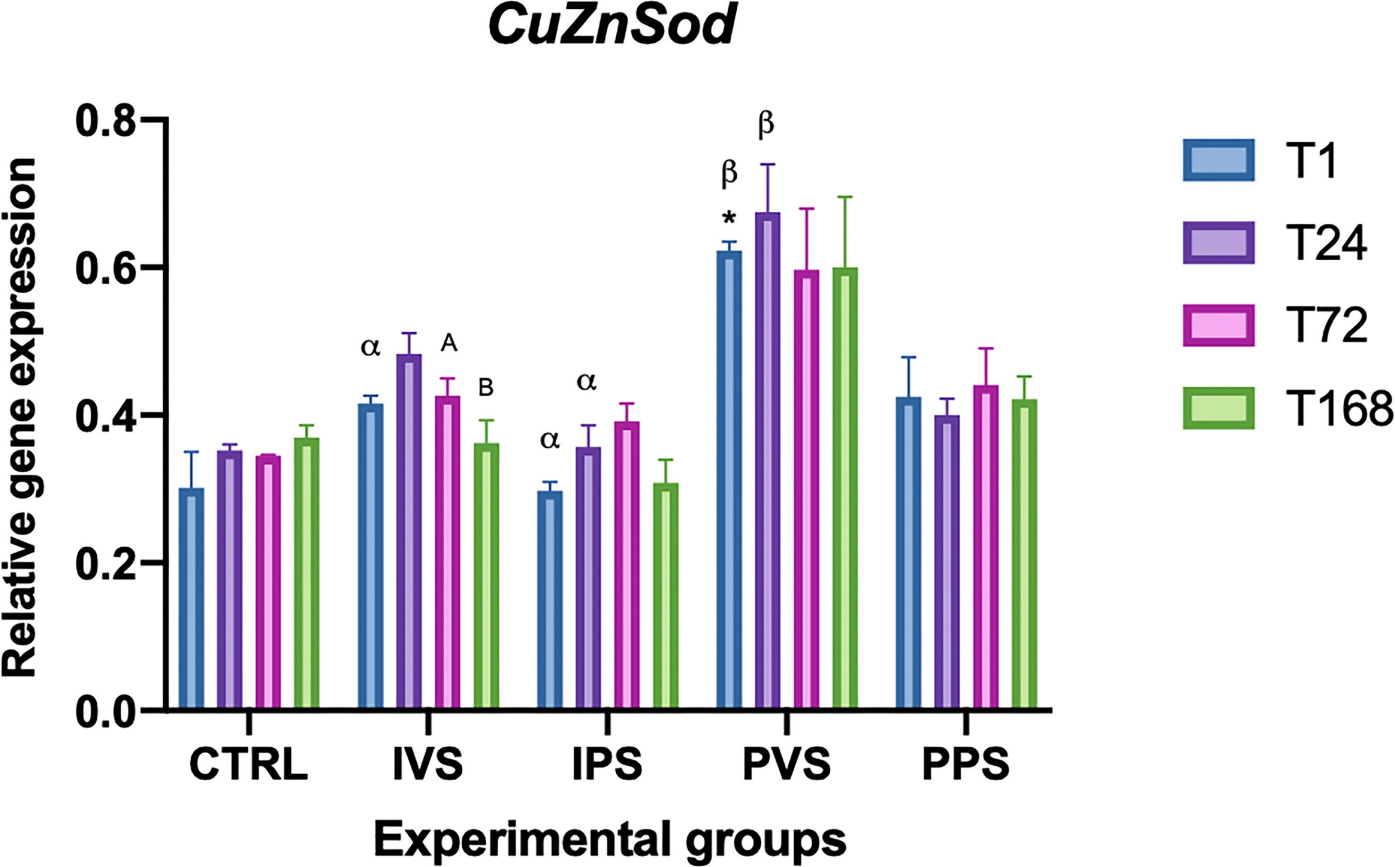
Relative expression of CuZnSOD mRNA in the spleen of *Sparus aurata* during experimental diet administration. Data are presented as mean ± SD (n = 5). Asterisks (*) indicate significant differences between each experimental group and the control group (CTRL) at the same time point. Letters are shown only when statistically significant differences are detected. Different capital letters indicate significant differences among time points within the same treatment group, whereas different Greek letters indicate significant differences among treatment groups at the same time point (*p* < 0.05). IVS, fish fed Imoviral^®^ and infected with *Vibrio anguillarum*; IPS, fish fed Imoviral^®^ and uninfected; PVS, fish fed commercial feed and infected with *Vibrio anguillarum*; PPS, fish fed commercial feed and uninfected; CTRL, control group.

Regarding CuZnSOD, the only experimental group that showed statistically significant differences with the control was PVS at T1(p<0.05). Considering each time point, significant differences were detected between IVS and PVS at T1, while IPS and PVS showed differences at T1 and T24 (p<0.05). Moreover, significant differences within the same experimental group were observed in the IVS group, between T72 and T168 (**Figure 8**).

**Figure 9:**
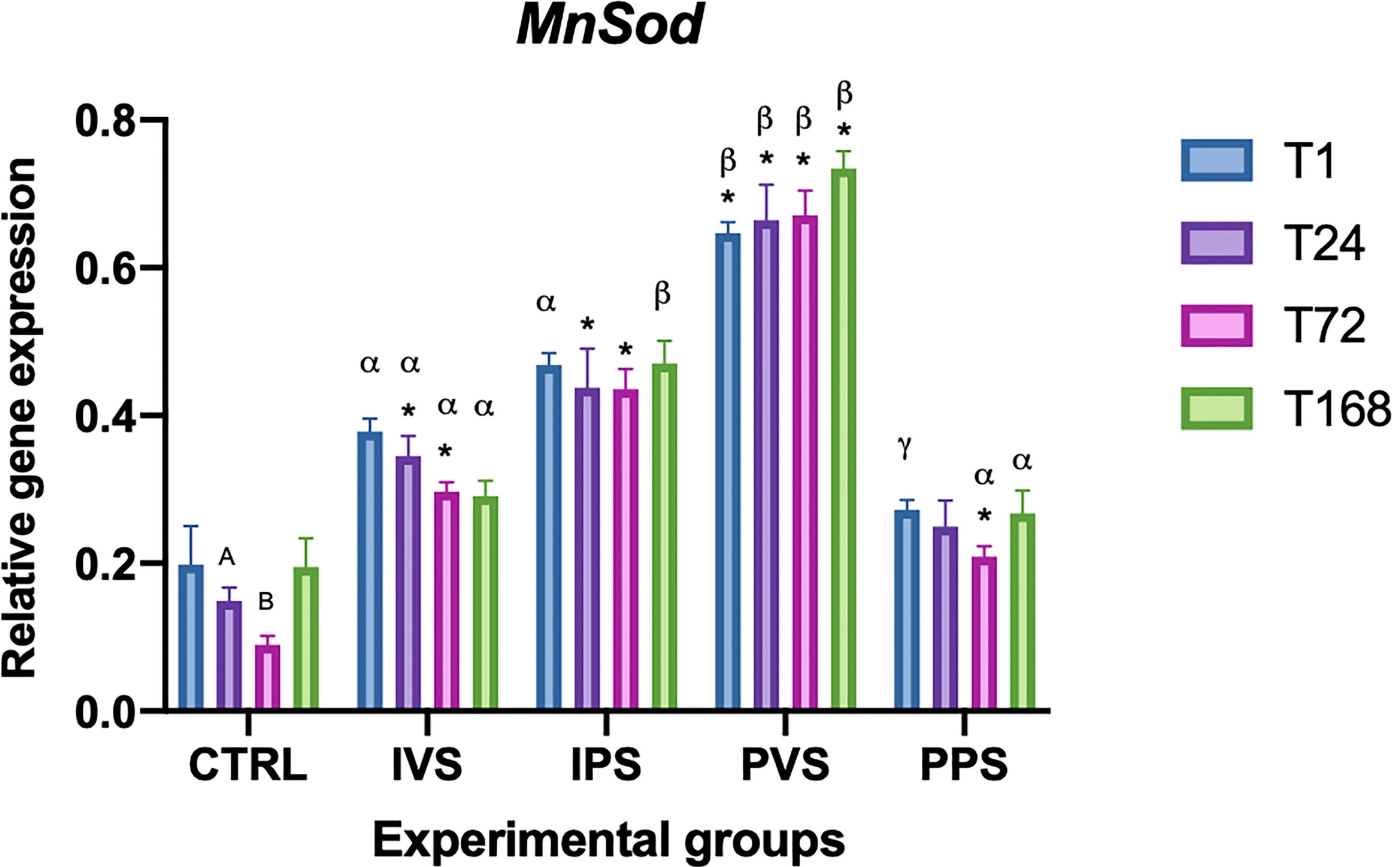
Relative expression of MnSOD mRNA in the spleen of *Sparus aurata* during experimental diet administration. Data are presented as mean ± SD (n = 5). Asterisks (*) indicate significant differences between each experimental group and the control group (CTRL) at the same time point. Letters are shown only when statistically significant differences are detected. Different capital letters indicate significant differences among time points within the same treatment group, whereas different Greek letters indicate significant differences among treatment groups at the same time point (*p* < 0.05). IVS, fish fed Imoviral^®^ and infected with *Vibrio anguillarum*; IPS, fish fed Imoviral^®^ and uninfected; PVS, fish fed commercial feed and infected with *Vibrio anguillarum*; PPS, fish fed commercial feed and uninfected; CTRL, control group.

The expression of MnSOD varied significantly between all experimental groups and CTRL, at almost all T analyzed (p<0.05). More specifically, both IVS and IPS group showed significant differences with controls at T24 and T72. The same was noted for PVS at each time point, while PPS group had significance only at T72. No differences were observed within the same experimental group. In contrast, significant differences were detected between the experimental groups at the same time point: both IVS and IPS groups showed significance compared with PVS and PPS at T1. Regarding the other treatment group, significant differences were detected at T24 between IVS and PVS (p<0.05), at T72 for both IVS and PPS group compared with PVS, and at T168 for both IVS and PPS group compared with PVS, as well as IPS compared with PPS (**Figure 9**). Control group showed a difference in expression between T24 and T72 (p<0.05).

### 3.4 Genes involved in cellular metabolism

**Figure 10:**
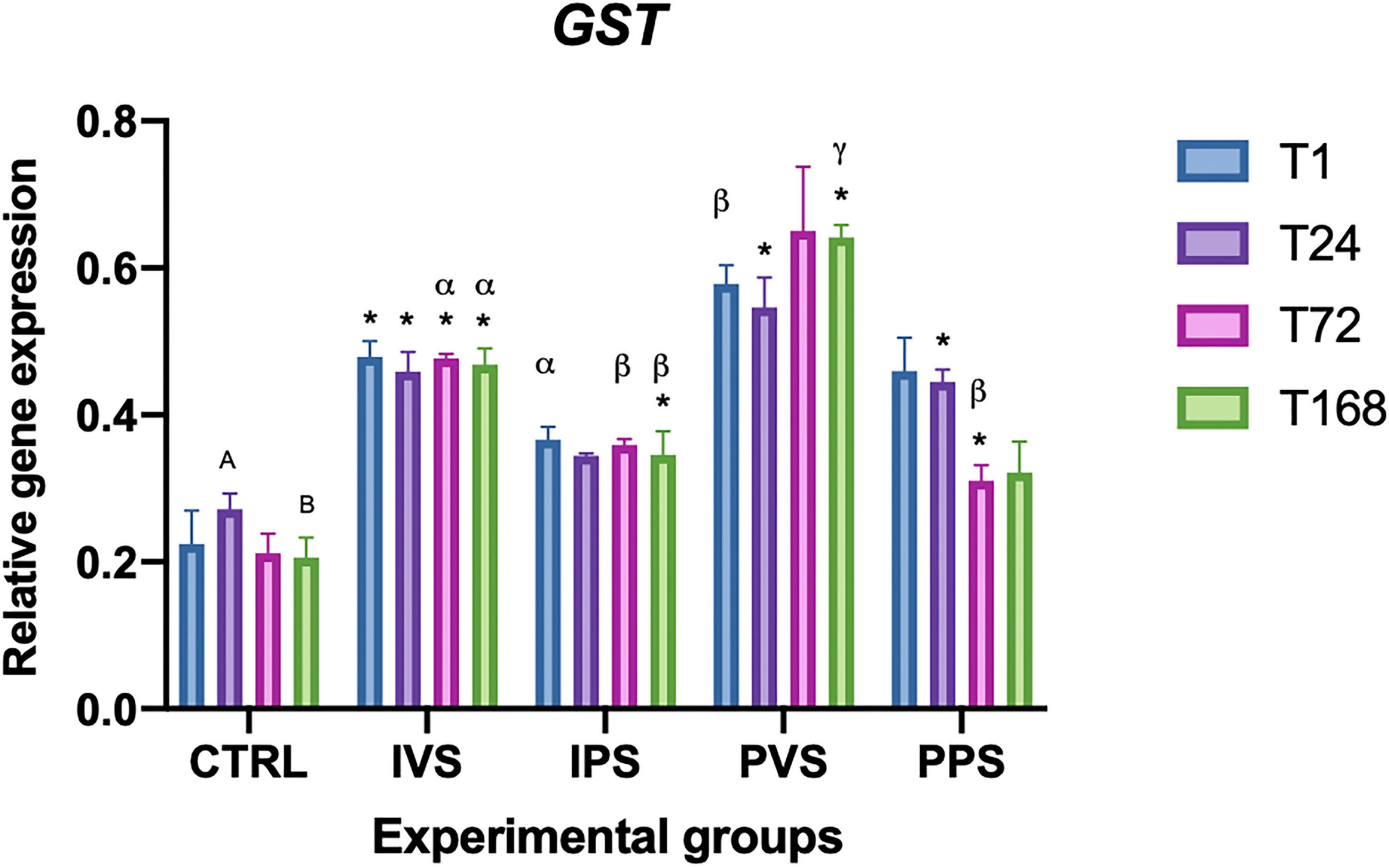
Relative expression of GST mRNA in the spleen of *Sparus aurata* during experimental diet administration. Data are presented as mean ± SD (n = 5). Asterisks (*) indicate significant differences between each experimental group and the control group (CTRL) at the same time point. Letters are shown only when statistically significant differences are detected. Different capital letters indicate significant differences among time points within the same treatment group, whereas different Greek letters indicate significant differences among treatment groups at the same time point (*p* < 0.05). IVS, fish fed Imoviral^®^ and infected with *Vibrio anguillarum*; IPS, fish fed Imoviral^®^ and uninfected; PVS, fish fed commercial feed and infected with *Vibrio anguillarum*; PPS, fish fed commercial feed and uninfected; CTRL, control group.

The expression of GST varied significantly between the CTRL and experimental groups (p<0.05) over time. In particular, the IVS group showed significance between every time point and control, unlike the IPS group which showed significant differences only at T168 (p<0.05). The PVS group was significantly different from control group at T24 and T168, while PPS group at T24 and T72 (p<0.05). No degrees of difference were observed within the same experimental group. Regarding the differences between experimental groups at the same time point, the IPS group showed significance compared with PVS at T1(p<0.05), while at T72 significant variations were noted for IVS *vs* IPS and PPS. Significant differences were also detected between IVS, PVS, and IPS, as well as between the IPS group and PVS at T168 (p<0.05) (**Figure 10**). Control group showed a difference in expression between T24 and T168 (p<0.05). Most Significant treatment differences comparing to time interactions are showed in S5.

## 4 Discussion

Over the past decade, immunostimulants have received extensive attention revealing potential beneficial effects against various pathogens, enhancing the immune response of fish, minimising the risk associated with the use of chemical agents (including antibiotics) and preventing damage caused by toxic compounds both in fish and humans. The use of natural extracts is highly recommended in aquaculture and considered as a safe, environment-friendly alternative approach to immunoprophylactic control (5, 25, 26). In the present study, gilthead sea bream fingerlings were fed with commercial pellets supplemented with complex Imoviral^®^ (25mg/10 gr pellet) in a four-week feeding trial. Selected innate immune response genes were then analysed in the spleen of both uninfected and *Vibrio anguillarum* - infected fish. The immunostimulant properties of the individual extracts of Imoviral^®^ have already been demonstrated on teleosts, except for blackcurrant (27–29). To our knowledge, this study represents the first investigation on the possible effect of Imoviral^®^ administration in teleosts as well as the potential synergistic effect of its natural extracts. The expression of ten genes involved in the acute phase response and oxidative stress were evaluated. These genes were chosen based on different criteria. Briefly, the proinflammatory cytokine genes *TNF-α* and *IL-1β* were selected due to their importance as inflammation markers (30). Hepcidin and defensin are antimicrobial peptides that mediate the innate immune response and show strong antimicrobial activity against *Vibrio anguillarum* in *Sparus aurata* (31, 32). CAT, CuZnSOD, and MnSOD are antioxidant enzymes involved in defense mechanisms against oxidative stress (33). The GST enzyme is also involved in the same processes, playing a crucial role in protecting cells against oxidative damage and detoxifying harmful substances (34). TGF-β is a regulatory cytokine with an anti-inflammatory function. However, it can also promote fibrosis and contribute to chronic diseases (35). IL-10 belongs to the same group of anti-inflammatory cytokines that regulate the immune response, playing a key role in maintaining immune homeostasis and preventing tissue damage caused by excessive inflammation (36). It has been shown that the administration of plant or fungal extracts can alter the transcription of pro- and anti-inflammatory cytokine genes in different organs (37). TNF-α and IL-1β are pro-inflammatory cytokines that show a generalized tendency to increase expression levels following the administration of natural immunostimulants such as *Salvia officinalis*, *Lippia citriodora*, fenugreek, and microalgae in several fish species including gilthead sea bream (37, 38).

Data obtained from this study demonstrated that the administration of Imoviral^®^ complex does not affect morphometrical and growth parameters.

Our results showed a modulation of gene expression dependent on different treatments and exposure times. The expression of antioxidant genes, such as catalase (CAT), CuZn superoxide dismutase (CuZnSOD), and manganese superoxide dismutase (MnSOD), showed temporal variations among the experimental groups. These findings highlight the dynamic nature of immune responses in fish under stressful conditions such as an infection with *Vibrio anguillarum*. In accordance with previous studies investigating the role of feed additives on immune status of fish, the levels of TNF-α were higher in the two groups fed with Imoviral^®^ (IVS and IPS) at T1, T24, and T72, both compared to the control group and compared to the groups fed only on commercial feed and injected with PBS (PPS) (37, 39). It is worth noting that our results showed a differential response in the modulation of TNF-α and IL-1β between the infected groups: IVS (fed with Imoviral^®^) and PVS (fed with commercial feed). As can be seen in **Figure 1**, *TNF-α* levels in IVS group are higher than CRTL at T1, T24 and T72; with a subsequent reduction demonstrating a strong initial response and then a regular expression decrease, probably due to the Imoviral^®^ effect. A different response was observed in PVS group where TNF-α expression showed an increasing trend from T1 to T168. This result contrasts with what was reported by Espinosa, Beltrán, Messina, & Esteban (2020) (40), where gilthead sea bream specimens, following the administration of *Jasonia glutinosa*, did not report significant changes in TNF-α expression. Expression levels of IL-1β (**Figure 2**) were higher in IVS (fed with Imoviral^®^ and infected with *V. anguillarum*) at T1 than in CRTL, while the IPS and PPS groups (fed with Imoviral^®^ and injected with PBS showed no significant changes compared to the CRTL group. Once again IVS and PVS showed a time-dependent trend in expression levels. IVS group showed a moderate but significant increase in *IL-1β* at 1 and 24 hpi while it was always up-regulated in PVS group, showing a peak of expression at 24 hpi. The IL-1β gene downregulation in the IVS experimental group compared to the PVS group indicates the potential of the immunostimulant Imoviral^®^ against the Vibrio infection, suggesting an impact on inflammatory pathways, aligning with the known bioactivity of *Uncaria tomentosa*, a key component of Imoviral^®^, which exhibits immunomodulatory and anti-inflammatory effects. Its mechanism involves reducing cytokine overexpression and oxidative stress, thereby preventing excessive inflammation. This suggests that Imoviral^®^ does not merely stimulate immunity but fine-tunes the inflammatory cascade, reinforcing its potential as a functional feed additive in aquaculture. A previous study found that the levels of IL-1β in specimens of gilthead sea bream fed with *Jasonia glutinosa* increased after 15 days from the start of the feeding trial but decreased after 30 days compared to CRTL (40, 41); in accordance with this study, we did not find any significant change in IL-1β, evaluated after 30 days feeding trial, in the IPS group compared to CRTL. In addition, it should also be noted that some of the extracts present in Imoviral^®^ have an anti-inflammatory activity, as in the case of *Uncaria tormentosa*, which may act by regulating TNF-α expression. Particularly, TNF-α expression showed modulation patterns consistent with immune regulation. This modulation may be linked to the synergistic effects of *Uncaria tomentosa* and beta-glucans present in Imoviral^®^. Beta-glucans are known to activate macrophages and enhance pathogen recognition, while *Uncaria tomentosa* reduces excessive cytokine signaling, collectively balancing pro-inflammatory responses (31). Anti-inflammatory and antibacterial activity that could modulate the pro-inflammatory response in a time-dependent manner should be more thoroughly investigated in further studies. The finding that IPS did not show moderate expression of TNF-α and did not show significant changes in IL-1β expression compared to CTRL suggests that Imoviral^®^ does not induce a significant immune response under basal conditions, but that it nonetheless provides protection in the case of infection and therefore for IVS. Proinflammatory cytokines are essential for mediating the inflammatory process and are produced by leukocytes activated in response to pathogenic signals. However, for *TNF-α* and other cytokines there seems to be a fine line between benefit and harm; a defence agent that is generally helpful in the local control of injury and infection may also be toxic when it is released in large amounts or in the wrong place (42). Furthermore, although not analysed in this study, it is necessary to evaluate the role of other genes associated with the anti-inflammatory response, which regulate and reduce the expression of pro-inflammatory cytokines when necessary, preventing collateral damage to host tissues and avoiding waste of bioenergetic resources (43). In the groups fed with Imoviral^®^, hep expression did not show relevant variations compared to the CTRL, except in the IVS group, where a significant decrease was observed at T168. Conversely, in the PVS group, hepc was consistently upregulated compared to both CTRL and IVS, with peak levels recorded at 72 and 168 hpi (**Figure 3**). Def levels significantly decreased in IPS compared to CTRL (at T1 and T24), while IVS also showed a significant difference from CTRL at T1. In both IVS and IPS, expression was strongly reduced compared to PVS, where the gene was highly expressed at all time points (**Figure 4**). The hepcidin and defensin genes are expressed in a wide range of tissues and generally exhibit upregulation after bacterial infection, therefore, the high levels of expression recorded in PVS are not surprising. Hepcidin expression varied between groups, indicating iron metabolism involvement. The observed regulation of hepcidin in IVS could be associated with IL-1β signaling and antioxidant-rich components such as blackcurrant extract in Imoviral^®^. These compounds mitigate oxidative stress and inflammation, indirectly influencing hepcidin expression and iron homeostasis during infection. The downregulation of antimicrobial peptide gene expression levels may depend on prolonged administration of the experimental diet (30 days) as previously observed. Furthermore, the low expression of hep may depend on the levels of IL-1β. It is known that hepcidin is greatly stimulated by inflammation, and is principally induced by interleukin-1, interleukin-6, and LPS (44), so it is possible that the low expression of IL-1β influenced the expression of this antimicrobial peptide. A more critical evaluation of the reduced hepcidin and defensin expression observed in Imoviral^®^-fed fish is also warranted. While the initial hypothesis associated this pattern with a controlled inflammatory response, it is equally plausible that the lower AMP transcription reflects a partial dampening of antimicrobial effector mechanisms. AMPs have been identified as being of crucial importance to the innate immune response at its earliest stage. The strong induction of these molecules in the PVS group, when contrasted with the attenuated response in the IVS group, could indicate a trade-off between reduced inflammatory activation and weaker late-stage antimicrobial capacity. This possibility cannot be excluded based solely on transcriptional data, and future work including pathogen load quantification or functional bactericidal assays will be essential to clarify whether Imoviral^®^ supplementation modulates AMP expression without compromising host defence.

The analysis of CAT expression showed a moderate increasing trend over time, with a significant peak at 72- and 168-hours post-infection (hpi) in the PVS group. Significant differences between experimental and control groups were found at different time points, particularly between IVS at 24 hours and PPS at 1 hour, as well as between PVS at 1, 24 and 72 hours. These different responses among experimental groups could be related to the efficacy of Imoviral^®^ in boosting the immune system, potentially leading to a more robust antioxidant response during the infection.

The significant variation in CAT levels at 24 hpi could be attributed to the initial acute phase of response, typically characterized by an increase in oxidative stress followed by a controlled reduction to prevent tissue damage (45). Regarding the CuZnSOD, no significant differences were found in the IPS group compared to controls. The expression of this gene was more variable among the experimental groups, with notable fluctuations at specific time points. The PVS group was the only one that exhibit significant differences between controls at the same time point (1 hpi). In addition, relevant differences were observed between IVS and PVS at 1 hpi, as well as between IPS and PVS at both 1 and 24 hpi. These data suggest that the PVS group might have a stronger oxidative response than the groups fed Imoviral^®^ or PBS alone. These results indicate that the complex formulation of Imoviral^®^ could induce a more balanced immune response, improving the ability of fish to cope with oxidative stress and the inflammatory process during infection (39, 46). Interestingly, at 72 hpi, the CuZnSOD expression did not differ significantly between groups, suggesting that the antioxidant capacity of the fish may return to baseline after the peak of inflammatory response. This effect, characterized by an initial increase of antioxidant responses followed by a decrease over time, was also observed in common carp infected by *Aeromonas hydrophila* (46, 47). The MnSOD gene also showed significant variations between all experimental groups and controls. More specifically, IVS and IPS groups differed significantly from controls at 24 and 72 hpi, while PVS showed significant differences at all time points. The PPS group exhibited significant differences only at 72 hpi. No significant differences were found within the same experimental groups over time. Regarding comparisons between experimental groups at the same time points, significant differences were observed between IVS and IPS compared to PVS and PPS at 1 hpi. At 24 hpi, IVS differed significantly from PVS; at 72 hpi, IVS and PPS differed from PVS; and at 168 hpi, IVS and PPS differed from PVS, while IPS differed from PPS. GST expression analysis showed significant differences between the experimental groups and the controls over time. In particular, GST transcript levels in IVS group showed significant differences at all time points compared to the control, while IPS showed significant differences only at 168 hpi, PVS group showed differences at 24 and 168 hpi, while PPS group at 24 and 72 hpi. Differences between experimental groups at the same time point were detected between IPS and PVS at 1 hpi and between IVS, IPS and PPS at 72 hpi. Different responses of GST in the IVS and PVS groups suggest that, although Imoviral^®^ may contribute to the regulation of oxidative stress, prolonged exposure could also be associated with changes in detoxification pathways (47, 48). While such modulation may reflect an adaptive response, alternative explanations should also be considered. Increased GST transcription may indicate a heightened detoxification demand associated with bacterial challenge or with the metabolic processing of dietary components, rather than representing solely an adaptive enhancement of antioxidant capacity. Similarly, GST upregulation may also reflect a prolonged cellular stress response, particularly under conditions characterized by sustained ROS production or increased xenobiotic load. Future studies integrating enzymatic assays or oxidative-damage markers will therefore be useful to better clarify the functional significance of GST modulation in Imoviral^®^-fed fish. Antioxidant enzymes (CAT, CuZnSOD, MnSOD, GST) exhibited differential expression trends. The upregulation of these enzymes in IVS may result from the presence of beta-glucans and shiitake mushroom extracts in Imoviral^®^, which enhance antioxidant defenses and reduce reactive oxygen species. This mechanistic link supports the hypothesis that Imoviral^®^ supplementation strengthens cellular resilience under pathogenic stress. Whilst the present results highlight the modulation of antioxidant-related genes, the lack of direct measurements of oxidative damage markers (e.g. lipid peroxidation or reactive oxygen species (ROS) levels) does not allow a direct evaluation of the oxidative status of the fish. Consequently, prospective studies integrating gene expression analyses with biochemical indicators of oxidative stress would facilitate further elucidation of the biological significance of the observed antioxidant responses. The interplay of cytokines and oxidative stress markers suggests a complex immune response. TNF-α modulation reflects the dual role of Imoviral^®^ components: beta-glucans stimulate innate immunity, while *Uncaria tomentosa* prevents hyperinflammation. Hepcidin regulation aligns with antioxidant activity from blackcurrant, reducing iron-driven oxidative damage. Enhanced antioxidant enzyme expression further confirms the contribution of shiitake-derived polysaccharides in maintaining redox balance. These mechanisms collectively demonstrate Imoviral^®^’s capacity to optimize immune and metabolic pathways during infection (49–53). The TgFb expression did not show statistically significant differences compared to controls, although variations were detected over time within some experimental groups, as in the case of IPS group at 24, 72 and 168 hours, and in the PVS group at 1 and 72 hours. In addition, significant differences between experimental groups were found at 1 hpi between PVS and PPS and between IVS, IPS and PPS compared with PVS. The absence of significant changes in TgFb expression suggests that supplementation with Imoviral^®^ does not directly affect this gene in the context of acute bacterial infection. The IL-10 expression showed significant differences over time within the same group, with marked variations between 1 and 168 hours in the IVS group and among all time points in the PVS group. Compared with controls, the IPS group showed differences at 1 and 24 hours, while PPS at 24 hours, and both IVS and PVS at 168 hours. Differences in gene expression levels at the same time point were significant at 72 hours between IPS and PVS, and between IPS and PPS compared to PVS. This suggests that the immunostimulant effect of Imoviral^®^ might improve the anti-inflammatory response, especially in prolonged infection, by promoting the expression of IL-10, a key anti-inflammatory cytokine (54).

## 5 Conclusion

In conclusion, our finding suggests that a diet supplemented with Imoviral^®^ complex may provide some immunomodulatory support in fish, enabling a faster immune response when challenged with a bacterial pathogen. This highlights the modulatory role of functional feeds in the acute phase immune response. The effect is further supported by the increased expression levels of target genes in the PVS group, which lacked the adjuvant activity of Imoviral^®^ against the pathogen. These results provide preliminary evidence of the potential of Imoviral^®^ as a valuable immunomodulant for farmed gilthead sea bream and as a possible alternative to prophylactic and therapeutic strategies based on vaccines and antibiotic. This study contributes to an increasing amount of research supporting the use of functional diets to improve fish health, welfare, and farm productivity in modern aquaculture. Nevertheless, further research is needed to fully elucidate the mechanisms and practical applications of Imoviral^®^, including the analysis of additional immune-related genes, assessment of anti-inflammatory and antibacterial activities, evaluation of different doses and feeding durations, testing in other farmed teleost species, and investigation of protective effects against a broader range of fish pathogens.

## Data Availability

The original contributions presented in the study are included in the article/**Supplementary Material**, further inquiries can be directed to the corresponding author/s.
