# Supplementary material for: Evaluation of Imoviral® effects on early immune response in Sparus aurata challenged with Vibrio anguillarum
Source: Front Immunol. 2026 Apr 23;17:1681468. doi: 10.3389/fimmu.2026.1681468 (PMC13151914; doi:10.3389/fimmu.2026.1681468)
Supplement: Supplementary file 1 [file DataSheet1.pdf]

Supplementary Material

Supplemental material 1. Primer sequences, amplicons size (bp), qPCR efficiencies (E%), and correlation coefficients (R^2) of the calibration

Primer	Forward primer sequence	Reverse primer sequence	Size (bp)	E (%)	R2
tnf-α	CTCACACCTCTCAGCCACAG	CAGTTTGTCGCCTCTGTTCA	186	109	0.99
il-1β	CTGCTCAACATCTTGCTGGA	TCGGACTAAGTGCCTCTGCT	135	90	0.99
hep	GCCATCGTGCTCACCTTTAT	CTGCTGCCATACCCCATCTT	152	98	0.99
def	AGGGCAATGATCCAGAAATG	CCGTGATGACCAACGATGTA	97	96	0.99
ef1-α	CTGTCAAGGAAATCCGTCGT	TGACCTGAGCGTTGAAGTTG	87	102	0,98
gap-dh	AGCCACTCCTCCATCTTTGA	TGCTGTAGCCGAACTCATTG	97	105	0.99
β-act	CACCGCAAATGCTTCTAACA	CTGAAGCCATCCCAATGAGT	149	100	0.95
18S	GACAAATCGCTCCACCAACT	CCTGCGGCTTAATTTGACTC	134	103	0.99
cat	TTCCCGTCCTTCATTCACTC	CTCCAGAAGTCCCACACCAT	80	112	0.96
CuZnSod	CCATGGTAAGAATCATGGCGG	CGTGGATCACCATGGTTCTG	164	98	0.99
MnSod	CCTGGGTCTGTGAGGTTGAA	CACACCTGCGTATTTGACCA	122	78	0.64
gst	AGAGGGAGAGAGACACGACT	GCCTGGATAAACGCACACAT	151	103	0.55
TgFb	AGACCCTTCAGAACTGGCTC	ACTGCTTTGTCTCCCCTACC	146	80	0.99
Il-10	GAGCGTGGAGGAATCTTTCAA	GATCTGCTGGATGGACTGC	105	100	0.99

Supplemental material 2. *Two-way ANOVA summary for gene expression data across experimental groups and time points.* The table reports the sum of squares (SS), degrees of freedom (DF), mean squares (MS), F-values (with numerator and denominator degrees of freedom), P-values, and the percentage of total variation (effect sizes) explained for each factor - Groups, Time points, and their interaction—for all analyzed genes (TNF, Il1b, Hepc, Def, CAT, CuZnSod, MnSod, GST, TgFb, and Il10

Gene	ANOVA table	SS	DF	MS	F (DFn, DFd)	P value	% of total variation
**TNF**	Groups	0,4746	4	0,1187	F (1,808, 3,617) = 3697	P<0,0001	55,26
	Time points	0,0338	3	0,01127	F (1,791, 3,581) = 42,26	P=0,0034	3,935
	Groups x Time points	0,3447	12	0,02872	F (1,284, 2,569) = 177,9	P=0,0019	40,13
**Il1b**	Groups	3,104	4	0,7761	F (1,092, 2,184) = 1263	P=0,0005	86,1
	Time points	0,03351	3	0,01117	F (1,844, 3,688) = 15,04	P=0,0172	0,9294
	Groups x Time points	0,4467	12	0,03723	F (1,474, 2,949) = 99,05	P=0,0021	12,39
**Hepc**	Groups	5,025	4	1,256	F (1,145, 2,289) = 463,6	P=0,0011	62,27
	Time points	0,6376	3	0,2125	F (1,644, 3,287) = 486,1	P<0,0001	7,901
	Groups x Time points	2,332	12	0,1943	F (1,913, 3,826) = 106,3	P=0,0005	28,9
**Def**	Groups	37,55	4	9,387	F (1,457, 2,915) = 8910	P<0,0001	82,54
	Time points	1,498	3	0,4995	F (1,079, 2,158) = 318,2	P=0,0022	3,294
	Groups x Time points	6,369	12	0,5308	F (1,882, 3,764) = 234,4	P=0,0001	14
**CAT**	Groups	1,375	4	0,3437	F (1,743, 3,486) = 431,1	P<0,0001	82,88
	Time points	0,04902	3	0,01634	F (1,124, 2,247) = 15,78	P=0,0477	2,955
	Groups x Time points	0,1749	12	0,01458	F (1,492, 2,983) = 7,790	P=0,0669	10,54
**CuZnSod**	Groups	0,6452	4	0,1613	F (1,722, 3,444) = 212,5	P=0,0003	83,17

	Time points	0,019	3	0,006333	F (1,971, 3,942) = 1,844	P=0,2718	2,449
	Groups x Time points	0,04219	12	0,003516	F (1,724, 3,448) = 2,003	P=0,2606	5,438
**MnSod**	Groups	1,962	4	0,4904	F (1,685, 3,370) = 458,8	P<0,0001	95,24
	Time points	0,02722	3	0,009075	F (1,029, 2,058) = 12,29	P=0,0698	1,322
	Groups x Time points	0,03516	12	0,00293	F (1,855, 3,709) = 3,382	P=0,1461	1,707
**GST**	Groups	0,9379	4	0,2345	F (1,181, 2,362) = 199,0	P=0,0024	87,61
	Time points	0,005708	3	0,001903	F (1,023, 2,045) = 3,362	P=0,2061	0,5332
	Groups x Time points	0,08354	12	0,006962	F (1,528, 3,056) = 6,134	P=0,0864	7,804
**TgFb**	Groups	1,618	4	0,4044	F (1,419, 2,837) = 128,3	P=0,0018	77,54
	Time points	0,1425	3	0,04751	F (1,403, 2,805) = 23,98	P=0,0181	6,832
	Groups x Time points	0,2277	12	0,01898	F (1,911, 3,821) = 7,886	P=0,0444	10,92
**Il10**	Groups	0,7836	4	0,1959	F (1,229, 2,458) = 117,3	P=0,0038	63,82
	Time points	0,1129	3	0,03763	F (1,140, 2,281) = 52,61	P=0,0127	9,194
	Groups x Time points	0,2768	12	0,02307	F (1,525, 3,050) = 15,88	P=0,0255	22,54

Supplemental material 3. Weight and length of fish during the trial

**Weight (g)**				
**Quarantine (Day 1-45); Imoviral adminisration day (46-75); Experimental infection (Day 68 - 75)**				
**Day 1**	**Day 45**		**Day 68**	**Day 75**
12.96 ± 0.94	17.41 ± 0.38	**IVS**	19.52 ± 0.38	20.38 ± 0.37
		**IPS**	19.52 ± 0.36	20.38 ± 0.37
		**PVS**	19.48 ± 0.38	20.37 ± 0.38
		**PPS**	19.50 ± 0.35	20.37 ± 0.35
		**CTRL**	19.48 ± 0.37	20.37 ± 0.37

Length (cm)				
Quarantine (Day 1-45); Imoviral adminisration day (46-75); Experimental infection (Day 68 - 75)				
Day 1	Day 45		Day 68	Day 75
10.56 ± 0.27	11.59 ± 0.1	**IVS**	12.11 ± 0.36	12.23 ± 0.36
		**IPS**	12.10 ± 0.37	12.22 ± 0.36
		**PVS**	12.09 ± 0.35	12.21 ± 0.36
		**PPS**	12.11 ± 0.37	12.21 ± 0.35
		**CTRL**	12.08 ± 0.39	12.20 ± 0.39

Supplemental material 4. Growth parameters

	Weight gain (WG)	Daily Growth Rate (DRG)	Specific Growth Rate (SGR)
**IVS**	7.27 ± 0.91	0.096 ± 0.01	0.59 ± 0.08
**IPS**	7.51 ± 1,11	0.1 ± 0.08	0.61 ± 0.1
**PVS**	7.4 ± 1.08	0.09 ± 0.01	0.6 ± 0.1
**PPS**	7.44 ± 1.09	0.09 ± 0.01	0.61 ± 0.11
**CTRL**	7.41 ± 0.99	0.09 ± 0.01	0.6 ± 0.1

Supplemental material 5. Significant Treatment × Time Interactions

Gene	Timepoints with Significant Differences	Groups Showing Differences	Description of Interaction / Trend

**TNF-α**	1, 24, 72 hpi	IVS vs CTRL; IVS vs PVS; PVS vs others	Early strong upregulation in IVS; opposite late increase in PVS; clear divergence over time.
**IL-1β**	1, 24, 72 hpi	IVS vs CTRL; IVS vs IPS; PVS vs all groups	IVS high only at 1 hpi; PVS highest overall with peak at 24 hpi and decline later.
**hepc**	1, 24, 72, 168 hpi	PVS vs CTRL; IVS vs IPS; IVS vs all at 168	Late strong upregulation in PVS; decline in IVS at 168 hpi; other groups stable.
**def**	1, 24, 72, 168 hpi	PVS vs all; IPS vs PPS; IVS vs IPS	PVS consistently high; IVS/IPS low early; PPS shows distinct mid-time response.
**Tgf-β**	24, 72, 168 hpi (within groups)	PVS vs PPS; IVS/IPS/PPS vs PVS at 24	Limited interactions; some within-group temporal changes.
**IL-10**	1, 24, 72, 168 hpi	IVS, PVS, IPS, PPS vs CTRL	Complex time-dependent shifts; strongest changes in IVS and PVS.
**CAT**	24, 72, 168 hpi	PVS vs CTRL; IVS vs CTRL; PVS vs PPS	PVS shows strong late peaks; IVS moderate; IPS stable.
**CuZnSOD**	1, 24 hpi	PVS vs CTRL; IVS vs PVS; IPS vs PVS	Mild interaction; early-time differences but

			limited temporal effects.
**MnSOD**	1, 24, 72, 168 hpi	All groups vs CTRL; IVS/IPS vs PVS/PPS	Strong, consistent between-group differences at nearly all times.
**GST**	1, 24, 72, 168 hpi	IVS vs CTRL; IPS vs PVS; PPS vs CTRL	Distinct time-response curves across groups; IVS always differing from CTRL.
